# Electrical Properties and Electromagnetic Interference Shielding Effectiveness of Interlayered Systems Composed by Carbon Nanotube Filled Carbon Nanofiber Mats and Polymer Composites

**DOI:** 10.3390/nano9020238

**Published:** 2019-02-10

**Authors:** Claudia Angélica Ramírez-Herrera, Homero Gonzalez, Felipe de la Torre, Laura Benitez, José Gerardo Cabañas-Moreno, Karen Lozano

**Affiliations:** 1 Programa de Doctorado en Nanociencias y Nanotecnología, CINVESTAV, Av. Instituto Politécnico Nacional 2508, Cd. de México 07360, México; caramirezh@cinvestav.mx (C.A.R.-H.); jcabanasm@cinvestav.mx (J.G.C.-M.); 2 Mechanical Engineering Department, The University of Texas Rio Grande Valley, 1201 West University Drive, Edinburg, TX 78539, USA; homero.gonzalez01@utrgv.edu; 3 Electrical Engineering Department, The University of Texas Rio Grande Valley, 1201 West University Drive, Edinburg, TX 78539, USA; felipe.delatorre01@utrgv.edu (F.d.l.T.); laura.benitez@utrgv.edu (L.B.)

**Keywords:** interlayered composites, forcespinning^®^, carbon nanofibers, carbon nanotubes, shielding effectiveness

## Abstract

The demand for multifunctional requirements in aerospace, military, automobile, sports, and energy applications has encouraged the investigation of new composite materials. This study focuses on the development of multiwall carbon nanotube (MWCNT) filled polypropylene composites and carbon nanofiber composite mats. The developed systems were then used to prepare interlayered composites that exhibited improved electrical conductivity and electromagnetic interference (EMI) shielding efficiency. MWCNT-carbon nanofiber composite mats were developed by centrifugally spinning mixtures of MWCNT suspended in aqueous poly(vinyl alcohol) solutions. The developed nanofibers were then dehydrated under sulfuric acid vapors and then heat treated. Interlayered samples were fabricated using a nanoreinforced polypropylene composite as a matrix and then filled with carbon fiber composite mats. The in-plane and through-plane electrical conductivity of an eight-layered flexible carbon composite (0.65 mm thick) were shown to be 6.1 and 3.0 × 10^−2^ S·cm^−1^, respectively. The EMI shielding effectiveness at 900 MHz increased from 17 dB for the one-layered composite to 52 dB for the eight-layered composite. It was found that the reflection of the electromagnetic waves was the dominating mechanism for EMI shielding in the developed materials. This study opens up new opportunities for the fabrication of novel lightweight materials that are to be used in communication systems.

## 1. Introduction

Conventional reinforced polymer composites have displayed potential for their use in aerospace, military, automobile, sports, and energy applications; however, the demand for multifunctional properties has encouraged the investigation of new materials. In recent years, numerous studies on the development of advanced polymer composites with improved electrical, mechanical, thermal, and electromagnetic interference (EMI) shielding properties have been conducted [1,2,3,4]. Carbon nanomaterials, such as carbon nanotubes (CNT) [5,6,7], carbon nanofibers (CNF) [8,9], and graphene [10,11] have been considered as ideal reinforcements for polymer composites due to their exceptional electrical and thermophysical properties coupled with their high aspect ratio, high strength, and stiffness, which leads to significant enhancements in electrical conductivity, mechanical stability, and EMI shielding. The properties of these attractive carbon-based systems have been for the most part independently evaluated. As the potential for practical applications is being reported, the development of systems with multifunctional properties has attracted considerable interest from the scientific community in synergistically improving thermal, electrical, and structural properties [12,13,14,15,16].

An innovative way to develop multifunctional composite materials is by developing interlayered composites that incorporate dissimilar architectures, mainly nonwoven CNF mats, woven carbon fibers (CF) fabrics, and CNT or CNF based buckypaper (BP) into polymer matrices using diverse fabrication methods. A wide variety of studies that focused on the electrical, mechanical, and electromagnetic interference shielding efficiency of interlayered composites have been reported [17,18,19,20,21,22,23,24,25]. For instance, Xu et al. [17] proposed a new method of depositing continuous CNT films onto CF fabrics using the floating catalyst chemical vapor deposition (FCCVD) method. The dry fabric was infiltrated into an epoxy resin and stacking twelve plies of the impregnated materials formed a laminate composite. They reported that the flexural strength and interlaminar shear strength of the composite containing 0.22 wt.% of CNT (per layer of carbon fiber fabric) increased by 16% and 21%, respectively, when compared to laminated composites without the added CNT. Moreover, the surface (in-plane) and volumetric (through-plane) electrical conductivity were also improved, 166% and 150%, respectively, upon the deposition of CNT (1.09 wt.%) on the CF fabrics.

Wang et al. [18] reported the preparation of BP/CF hybrid composites by adding BP between the CF plies using CF prepregs. Both, the surface and volumetric electrical conductivities exhibited significant improvements upon the addition of 7.99 wt.% of CNT. In another study, the CNT/CF/poly(ether-ether-ketone) (PEEK) multiscale composites that were prepared by directly spraying acid-treated CNT onto CF/PEEK prepregs were developed [19]. The resultant prepreg layers were stacked and consolidated by compression molding. The laminated composites showed enhanced mechanical properties with the presence of 0.5 wt.% of CNT, which was attributed to the mechanical anchoring effect of the CNT, improving fiber-matrix interactions. The electrical conductivity of the composites was also improved by the formation of the typical conductive network.

Several studies have also focused on the EMI shielding of these composites, for example, Park et al. [23] reported the preparation of laminates using multiple layers of BP with different stacking sequences and employing two types of dielectric materials (epoxy resin and polyethylene (PE)). BP/PE single-layer composites exhibited shielding effectiveness (SE) within the 20–60 dB range, and the increases were proportional to the conductivity that was recorded by the original BP used. The increase in the number of BP conducting layers and dielectric material coupled with the architecture of the system increased the electromagnetic interference shielding effectiveness (EMI SE) from 45 dB to close to 100 dB. In another study, Silveira et al. [24] developed multifunctional composites that were based on epoxy resin/glass fiber woven fabric prepregs and non-woven CF/Ni veils using compression molding. The developed laminated composites resulted in a high reflection of incident microwaves, being 91.4–100%. Composites of continuous carbon fibers (CCF) that were arranged on poly(ethylene terephthalate) (PET)-nonwoven fabric substrates using different orientations, and the number of layers have also been studied [25]. At high frequency (750 MHz–1.5 GHz), the orientation of the CCF showed a greater influence on the shielding performance than the number of layers. A three-layer composite with orientations between layer and layer of 0°–0°–45° reached the highest value of SE, 60 dB at 1.0 GHz.

Existent literature shows important improvements in the search to find materials with promising practical applications where the needed properties, such as electrical conductivity, shielding of electromagnetic interference, structural stability, and lightweight, are adequately complemented with the ease of fabrication and cost-effectiveness. The above-mentioned systems are highly desired in important applications, for example, in the development of rigid wall shelters for defense-related applications and in novel cable systems (uses ranging from everyday applications to aerospace applications). This study effectively addresses the needs that are mentioned above by developing nonwoven carbon composite mats, taking advantage of the Forcespinning^®^ method that uses centrifugal force to spin fine fibers (nano-, submicron-, and single digit fibers) at industrial scales, (hundreds of meters per minute) [26,27]. The developed multiwall carbon nanotube (MWCNT) filled carbon nanofiber mats were stacked using a matrix that was composed of MWCNT reinforced polypropylene. The morphology, electrical properties, and EMI SE of the developed composites were evaluated.

## 2. Materials and Methods

### 2.1. Materials

Poly(vinyl alcohol) (PVA) (Mw ≈ 85,000–124,000), with a viscosity of 27 cP and hydrolysis grade of 96%, was obtained from Kuraray, Inc., (Houston, TX, USA) and was used as received. Commercial grade polypropylene, PP 4280 W Impact copolymer (supplied by Total Petrochemicals, Houston, TX, USA), with a melt flow index = 1.3 g/10 min, was used as the matrix. MWCNT (cat. number 773840 from Sigma-Aldrich, St. Louis, MO, USA), with a purity ≥98%, average outside diameter of 10 nm, and length of 3–6 μm were used as one of the electrically conductive fillers. Sulfuric acid (reagent grade) was purchased from Sigma-Aldrich.

### 2.2. Production of MWCNT Filled Carbon Nanofiber Mats Using the Forcespinning^®^ Method

Solutions containing PVA and MWCNT in distilled water were prepared. The MWCNT (0.05 and 0.1 wt.%) were dispersed in distilled water in an ultrasonic bath for 15 min, and were then mechanically agitated for 15 min. Subsequently, the PVA (10 wt.%) was added to the MWCNT dispersion. The resulting solutions were subjected to magnetic stirring at 75 °C for 2.5 h in an oil bath. The solutions were maintained under magnetic stirring at room temperature for 24 h and then subsequently fed into a two-nozzle spinneret and spun at 9000 rpm for 3 min using the Cyclone L-1000 M from Fiberio Technology Corp. (Mission, TX, USA). The obtained nanofibers were collected in 11 cm × 11 cm hollow metal frames. This procedure was repeated until the desired thickness, based on grams per square meter (GSM), was obtained. The collected mats were dehydrated by exposure to sulfuric acid vapors, the mats were placed 5 cm above a heated bath of sulfuric acid whose temperature varied from 180 up to 280 °C. The treated mats were subsequently washed with distilled water to remove the remaining acid and then dried overnight at room temperature. The samples were then subjected to a carbonization process that consisted of a stabilization procedure at 240 °C for 15 min in air atmosphere, followed by ramping up to 1000 °C at a heating rate of 3 °C min^−1^ under a nitrogen atmosphere [28]. The areal density for the developed MWCNT reinforced carbon nanofiber nonwoven mats was 42 GSM.

### 2.3. Preparation of Nanoreinforced Polymer Composite Sheets (NRPCS)

Polypropylene pellets were mixed with MWCNT (15 wt.% or 7.07 vol.%) at 190 °C, 90 rpm for 30 min using a HAAKE Rheomix mixer on a HAAKE Rheocord Torque Rheometer (Thermo Fisher Scientific Inc. (Karlsruhe, Germany). After mixing, the composite was compression molded at 200 °C to form sheets of 0.1 mm in thickness. The NRPCS were used as the matrix for the MWCNT filled carbon nanofiber mats.

### 2.4. Fabrication of Interlayered Composites (IC)

**Figure 1:**
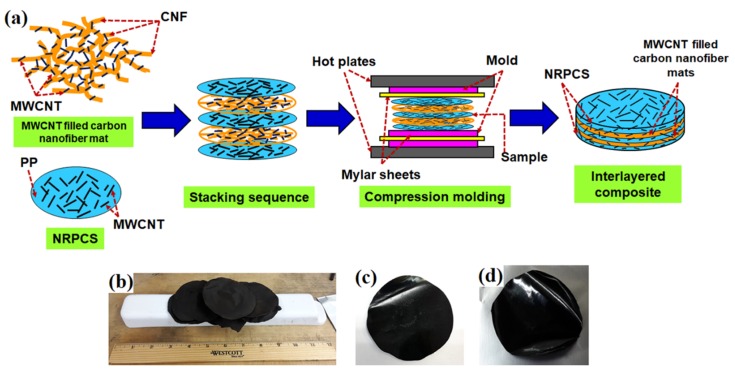
(**a**) Schematic representation of the fabrication process of interlayered composites; Digital pictures of (**b**) MWCNT filled carbon nanofiber mats; (**c**) NRPCS used as starting materials; and, (**d**) a resultant one-layer interlayered flexible composite.

Figure 1a depicts the schematics of the process that was used to fabricate the interlayered composites. Figure 1b shows digital images of the spun, dehydrated, and carbonized fiber mats. Figure 1c shows images of the MWCNT reinforced polymeric composite (NRPC), while Figure 1d shows a one-layer MWCNT filled carbon nanofiber mat with one layer of NRPCS. As shown in Figure 1a, MWCNT filled carbon nanofiber mats (0.1 mm thick) and NRPCS were cut in circles of 3 cm in diameter and then stacked into a metal mold. The lay-up sequence consisted in alternating one to eight layers of the MWCNT filled carbon nanofiber mats between the layers of the polymer composite sheets. The stacked multilayers were compression-molded at 200 °C and 42 MPa for 10 s. To prevent the composite from sticking to the compression mold, Mylar sheets were placed on each side of the sample. The final thicknesses of the interlayered composites were 0.1, 0.16, 0.3, and 0.65 mm for the systems with one, two, four, and eight layers, respectively. Table 1 shows a summary of the overall characteristics for the developed samples.

As can be observed from Table 1, the density of interlayered composites diminishes as the number of layers of MWCNT filled carbon nanofiber mats is increased. Additionally, it is noted that by increasing the number of layers, the total content of carbon matrix and local content of MWCNT is augmented, whereas the total content of PP diminishes given that the volume that is occupied by the carbon nanofiber mats is higher than the NRPCS in the interlayered systems. The interlayered composite with the higher numbers of layers (eight) is composed of 18 wt.% (43 vol.%) carbon content, 12 wt.% (9 vol.%) MWCNT, and 70 wt.% (48 vol.%) of PP.

### 2.5. Characterization

The morphology of the samples was analyzed while using a field-emission scanning electron microscope (FE-SEM) under an acceleration voltage of 1.0 kV (Sigma VP, Carl Zeiss, Jena, Germany). A statistical analysis of fiber diameter was conducted using Image J software, k1.45 version, National Institutes of Health (NIH), Bethesda, MD, USA. At least ten different SEM micrographs were used to determine the fiber diameters from at least 100 fibers. The mean fiber diameter and the histograms were generated using Minitab^®^ 17 Statistical Software (State College, PA, USA). Measurements of the surface resistivity of MWCNT filled carbon nanofiber mats, and interlayered composites on circular specimens of 3 cm in diameter and thicknesses from 0.1–0.65 mm, were obtained while using an R-CHEK RC2175 four-point probe surface resistivity meter (EDTM, Inc., Toledo, OH, USA) at room temperature. The average value of ten measurements at different locations in each sample was taken as the sheet resistance (*R*_s_, in Ω/sq) of the composite. Values of in-plane electrical conductivity (*σ*_i_, in S·cm^−1^) were obtained from the sheet resistance and the thickness (*t* in cm) of the sample, as shown in Equation (1):

(1) $$\sigma_{i}=\frac{1}{R_{s}t}$$

The through-plane electrical conductivity (*σ_t_*, S·cm^−1^) was determined by pressing the samples in between cooper electrodes with an effective area of 2.04 cm^2^ and using the Equation (2):

(2) $$\sigma_{t}=\frac{t}{AR}$$

where *t* is the thickness of the sample (cm), *A* is the effective area of the measuring electrodes (cm^2^), and *R* is the measured resistance of the sample (Ω).

**Figure 2:**
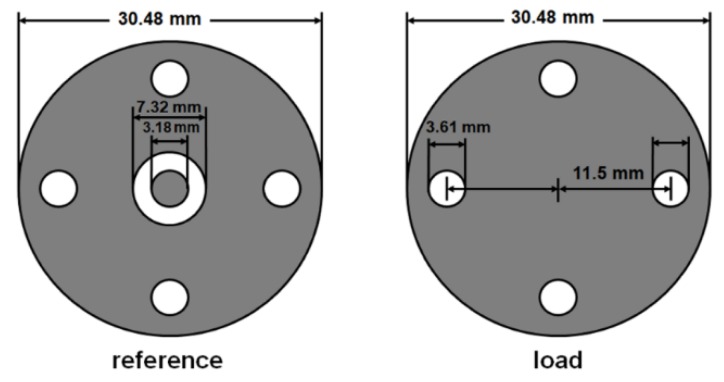
Fixture configuration for testing of electromagnetic interference (EMI) shielding effectiveness [29].

The EMI SE was measured at room temperature using an in-house manufactured coaxial flange fixture [29] that was connected to a Hewlett-Packard 8712C network analyzer (Palo Alto, CA, USA) that operates in a frequency test range of 0.3 to 1300 MHz. The measurements were conducted following a procedure that was described by Vasquez et al. [29]. The measurements require two samples: a reference specimen and a load specimen. Figure 2 shows the dimensions of the reference and load specimens. Each specimen was held between the two coaxial flanged fixtures and the values that were obtained from the analyzer were given in dB. The total EMI SE is the ratio of incident to transmitted energy, which can be expressed as [30,31,32,33,34]:

(3) $$SE_{T}=10\log\frac{P_{1}}{P_{2}}$$

where *P*_1_ is the received power density with the material present (load specimen) and *P*_2_, is the received power density without the material present (reference specimen).

The total experimental SE (*SE*_Total_) is the sum of the shielding by reflection (*SE*_ref_) and absorption (*SE*_abs_), involving the contribution of multiple reflections (*SE*_mr_), which can be obtained based on experimental parameters, as shown [31,32,33]:

(4) $$SE_{\mathrm{Total}}=SE_{\mathrm{ref}}+SE_{\mathrm{abs}}+SE_{\mathrm{mr}}$$

(5) $$SE_{\mathrm{ref}}=-10\log\left(1-R\right)$$

(6) $$SE_{\mathrm{abs}}=-10\log\left(1-A\right)=-10\log\left(\frac{T}{1-R}\right)$$

where *R*, *A*, and *T* represent reflectance, absorbance, and transmittance coefficients, respectively. These can be described in terms of scattering parameters (*S*-parameters), as obtained from testing, as follows:

(7) $$T={\left|S_{12}\right|}^{2}={\left|S_{21}\right|}^{2}$$

(8) $$R={\left|S_{11}\right|}^{2}={\left|S_{22}\right|}^{2}$$

(9) A=1−R−T

where |*S*_12_|^2^ (|*S*_21_|^2^) and |*S*_11_|^2^ (|*S*_22_|^2^) represent the power that is transmitted from port 1 to port 2 and vice versa, and the reflected power in both ports, respectively. The scattering parameters were obtained using the same set up described above for EMI SE measurements, but for these, only the load specimen was required.

## 3. Results

### 3.1. Morphological Analysis of Nonwoven Fiber Mats, MWCNT-Carbon Nanofiber Composite Mats, and Interlayered Composites

**Figure 3:**
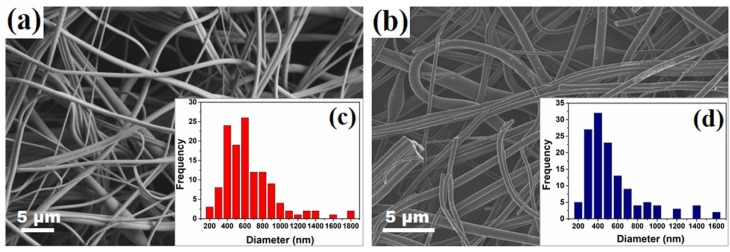
SEM images of (**a**) nonwoven Poly(vinyl alcohol) (PVA) nanofibers mat and (**b**) nonwoven carbon nanofibers (CNF) mat without MWCNT. Histograms representing fiber diameter distributions are shown as insets for both systems (**c**) and (**d**).

The microstructural analysis and fiber diameter distributions of spun nanofibers from solutions containing 10 wt.% PVA without the addition of MWCNT and the corresponding CNF mat obtained after the dehydration and carbonization processes are illustrated in Figure 3. Figure 3a depicts the spun PVA nanofibers, showing long continuous nanofibers with homogeneous surfaces and diameters that are mostly within the 400–600 nm range (Figure 3c). Figure 3b shows the developed carbon nanofibers after exposure to dehydration by the sulfuric acid vapor and subsequent heat treatment. The average carbon fiber diameter after heat treatment was 580 nm.

**Figure 4:**
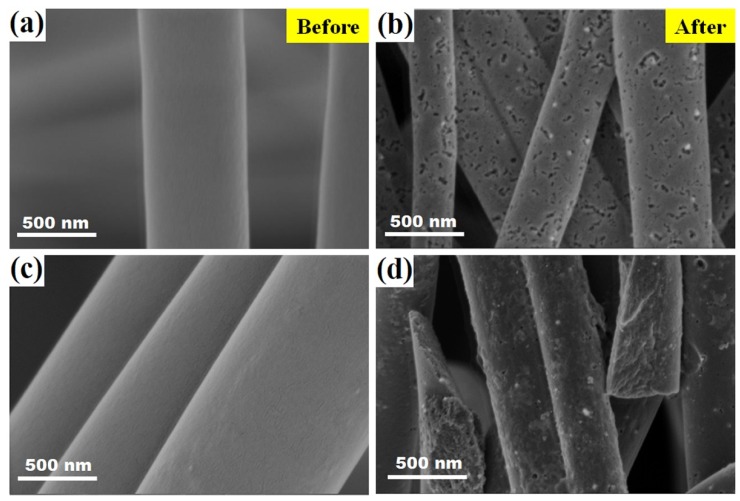
SEM images of MWCNT filled carbon nanofiber mats with different contents of MWCNT before and after carbonization. (**a**,**c**) PVA nanofibers mats with 0.05 and 0.1 wt.% MWCNT and (**b**,**d**) CNF mats filled with 0.05 and 0.1 wt.% MWCNT, respectively.

Figure 4 shows a comparison of the SEM images of the MWCNT filled carbon nanofiber mats with different MWCNT contents, before and after the carbonization process. It can be observed that the surface structure of the hybrid nonwoven nanofibers before the carbonization process appear to be homogeneous, with surfaces free of defects (Figure 4a,c). After the heat treatment, the surface of the fibers is significantly altered, as seen in Figure 4b,d. It is clearly observed that the nanofibers exhibit a rougher surface when compared to the pure PVA nanofibers and carbonized PVA fibers; and, the addition of the MWCNT resulted in a heterogeneous surface upon carbonization. Voids are clearly observed in the carbon nanofibers, which became more notable as the MWCNT content was augmented. During the initial formation of the fibers, solvent that is trapped in between MWCNT does not completely evaporates, upon carbonization, solvent or even not carbonized polymer leaves the system, leaving behind a porous surface.

**Figure 5:**
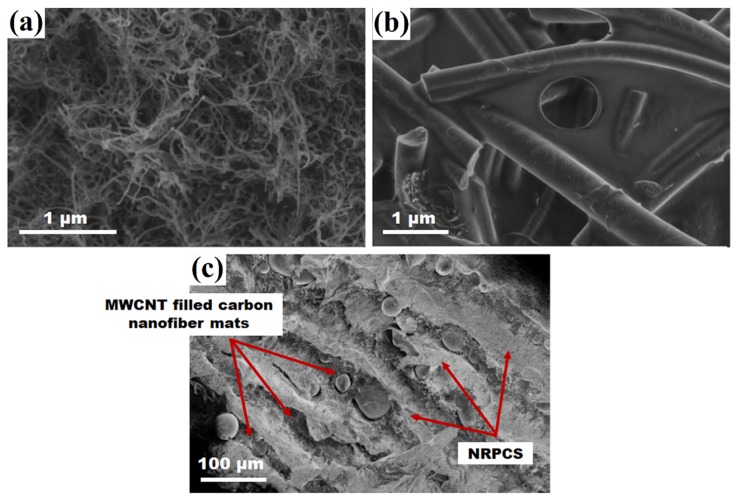
SEM images of (**a**) a cross-sectional of the NRPCS; (**b**) MWCNT filled carbon nanofiber mat embedded within the NRPCS; and, (**c**) the cross-sectional area of an eight-layered flexible composite.

SEM micrographs of some of the developed polymer composites are shown in Figure 5. As observed, the NRPCS that was prepared from PP and MWCNT exhibits a homogeneous distribution of MWCNT, lacking large agglomerates (Figure 5a). This helps in promoting the formation of a conductive network that is needed for percolation. In the same way, when the hybrid nonwoven CNF mats were embedded with the NRPCS (Figure 5b), it is clear that the polymer resin wets the developed CNF mats. Figure 5c shows the cross-sectional area of an eight-layer composite. This sample is composed of eight MWCNT filled carbon nanofiber mats pressed in between layers of NRPCS. The MWCNT filled carbon nanofiber mats can be distinguished by observing the darker areas running almost parallel to one another; these layers appear intercalated among the layers of NRPCS.

### 3.2. Electrical Properties of MWCNT Filled Carbon Nanofiber Mat and Interlayered Composites

The in-plane electrical conductivity of MWCNT filled carbon nanofiber mats and interlayered composites were analyzed. Table 2 shows the effect of the addition of MWCNT to CNF. As it can be observed, the CNF mat without MWCNT exhibited a value of electrical conductivity of ~1 S·cm^−1^, while the addition of 0.1 wt.% of MWCNT led to a slight increase of the in-plane electrical conductivity to 2.8 S·cm^−1^ (0.36 Ω·cm in electrical resistivity). The in-plane electrical conductivity of the NRPCS and the composite in the one layer of nonwoven CNF mats containing 0, 0.05, or 0.1 wt.% of MWCNT shows continuous increments as the MWCNT content is increased. The interlayered composite containing one layer of CNF mat filled with 0.1 wt.% of MWCNT shows a value that is three times higher (4.5 S·cm^−1^) than the reference sheet (NRPCS) and a factor of 1.5 higher than the composite using the CNF mat without MWCNT.

Adding more layers and increasing the content of MWCNT within the carbon nanofiber mat increases the electrical conductivity. The in-plane electrical conductivity of the composite with 0.1 wt.% of MWCNT within the carbon nanofiber mats increased from 4.5 up to 6.1 S·cm^−1^ (0.23 to 0.16 Ω·cm in electrical resistivity) for the interlayered composite containing eight layers. The increase in electrical conductivity of the multilayered systems is not significantly depicted in the in-plane conductivity measurements. Through-plane conductivity analysis, additional information that is especially related to these interlayered composites is provided. As the layers of MWCNT-carbon nanofiber composite mats and NRPCS increase, the through-plane conductivity increases, as depicted in Table 2, which summarizes the surface and volume electrical conductivity. It is clearly noted that, in all cases, the volume electrical conductivity is lower than the corresponding surface conductivity by at least by two orders of magnitude. The maximum value of volume electrical conductivity reached 3 × 10^−2^ S cm^−1^ in the eight-layered flexible composite.

### 3.3. EMI Shielding Effectiveness

According to the literature, the obtained electrical conductivity values are considered low to satisfy EMI shielding related applications [35,36,37]; though, electrical conductivity is not the absolute criterion to shield EMI [4,38]. EMI is attenuated by three major mechanisms: reflection, absorption, and multiple reflections. For reflection of the radiation from the surface of the shield, the shield must have mobile charge carriers to interact with the incoming electromagnetic field. Consequently, the shielding material tends to be electrically conductive [4,30,31,36,39]. Absorption is related to the existence of electric and/or magnetic dipoles in the shielding material, which interacts with the electromagnetic field. Multiple reflections refer to the reflections at various surfaces or interfaces within the shield, which are typically observed in systems that are loaded with fillers of large surface area, and therefore high interfacial area within the overall structure [4,32,36]. When the shielding by absorption is higher than 10 dB or when the distance between the reflecting surfaces or interfaces is large when compared to the skin depth, multiple reflections can be ignored, as it is often observed in metallic systems [4,31,33,36].

**Figure 6:**
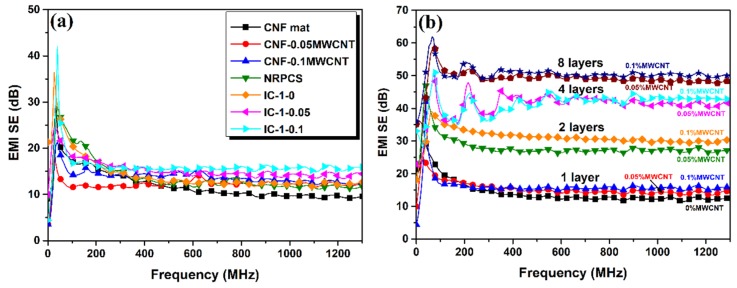
(**a**) Total experimental EMI shielding effectiveness (EMI SE) of nonwoven CNF mat, NRPCS, MWCNT filled carbon nanofiber mats with different MWCNT contents, and one-layered flexible composites; and (**b**) EMI SE of interlayered composites formed by different number of layers of MWCNT filled carbon nanofiber mats with variable MWCNT concentration.

The total experimental EMI SE versus frequency of the developed samples is shown in Figure 6a. The nonwoven CNF mat displays an SE value of ~12 dB, upon the addition of MWCNT (0.05 and 0.1 wt.%), a slight increase is obtained, reaching a value of 14 dB. The nanoreinforced polymer composite shows similar SE values. The combination of one layer of nonwoven MWCNT filled carbon nanofiber mat with NRPCS shows a slight increase to 17 dB (thickness of 0.10 mm) at 900 MHz (Figure 6a). Upon the addition of more layers, the SE increased considerably (Figure 6b). A two-layer composite (IC-2-0.1) almost doubled its SE, to 31 dB. The eight-layered flexible sample composed of nonwoven 0.1 wt.% MWCNT filled carbon nanofiber mats (IC-8-0.1) shows an ability to shield >50 dB at the tested frequencies within the *S*-band region.

On the other hand, for lightweight shielding materials, the specific SE (SSE, in dB·cm^3^·g^−1^), defined as SE divided by density (in g·cm^−3^), is also a critical parameter [40,41]. SSE provides accurate EMI SE information by considering the density, *ρ*, of the material (see Table 1), where a thinner material or one with lower density might exhibit higher EMI SE [41,42,43]. When considering that the SSE does not give thickness-based information, the absolute shielding effectiveness (SSE/*t* in dB·cm^2^·g^−1^) is used to evaluate the relationship between SSE and thickness. Table 3 shows the values of EMI SE, SSE, and SSE/*t* of each produced composite. In the *S*-band region, the nonwoven CNF mat without the incorporation of MWCNT shows a value of SSE of 36.1 dB·cm^3^·g^−1^, which is increased to 48.9 dB·cm^3^·g^−1^ with the addition of 0.1 wt.% of MWCNT. In the interlayered composites containing one and two layers of CNF mats, a significant decrease of the SSE was recorded, and then the values of SSE were augmented as the number of layers increased. A maximum value of SSE of 76.5 dB·cm^3^·g^−1^ was obtained for the IC-8-0.1 composite, which displayed the lowest density of the interlayered composites that were produced in this work (0.68 g·cm^−3^). Similarly, the SSE/*t* values show increases with the addition of MWCNT to the CNF mats, reaching a maximum value of 5433.3 dB·cm^2^·g^−1^. The interlayered composites exhibit lower values than those of the CNF mats. These values were highly increased in the two-layered composite. The increase in the thickness of the interlayered samples from 0.3 to 0.65 mm in IC-4-0.1 and IC-8-0.1 composites caused a remarkable decrease in the SSE/*t* values, recording a minimum value of 1176.9 dB·cm^2^·g^−1^ in the IC-8-0.1 composite. It can be noted that the increase in the thickness of these samples is mainly due to the presence of a significant amount of NRPCS in the interlayered structures. As it is well known, the PP matrix that is used to fabricate the NRPCS is transparent to the electromagnetic interference, exhibiting values of attenuation close to 0 dB [42,44], prompting the decrease in the SSE/*t* values in these composites.

**Figure 7:**
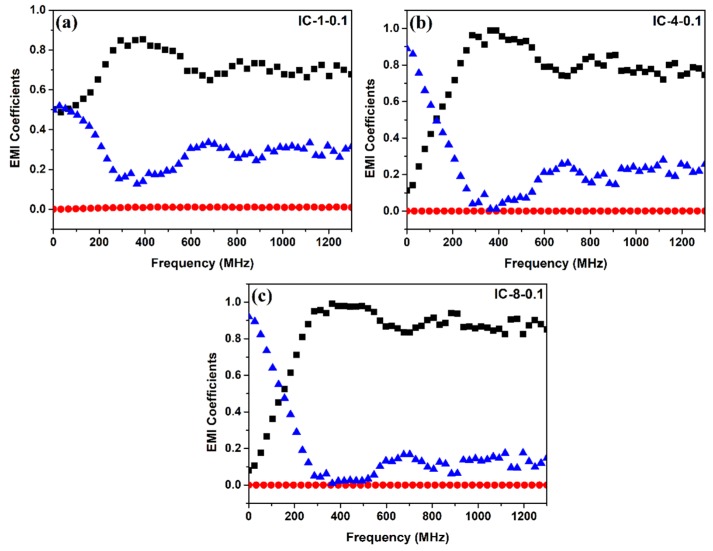
EMI power coefficients of reflectance (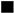), transmittance (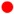), and absorbance (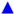) of the interlayered composites: (**a**) IC-1-0.1; (**b**) IC-4-0.1; and, (**c**) IC-8-0.1.

**Figure 8:**
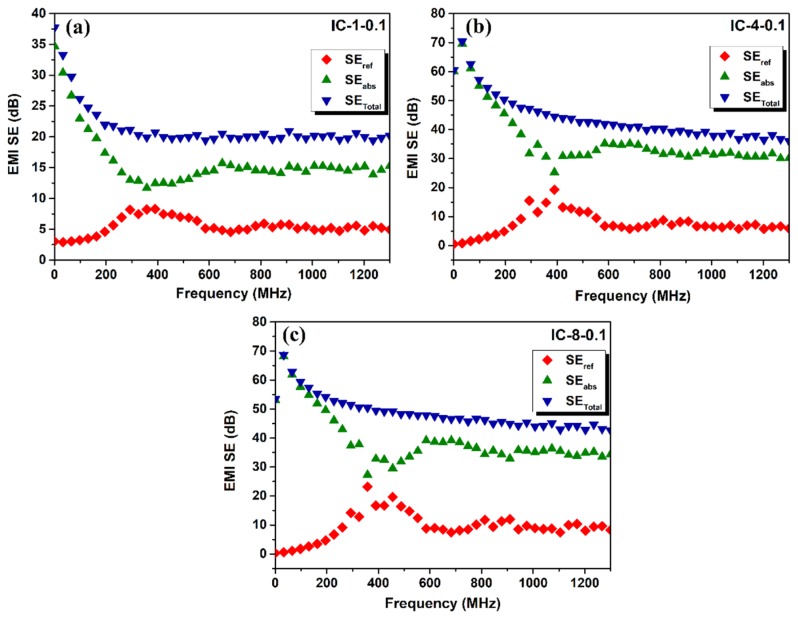
Reflection, *SE*_ref_ (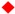), absorption, *SE*_abs_ (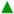), and total SE, *SE*_Total_ (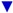) for interlayered composites: (**a**) IC-1-0.1; (**b**) IC-4-0.1; and, (**c**) IC-8-0.1.

The reflectance (*R*), transmittance (*T*), and absorbance (*A*) power coefficients for selected samples (IC-1-0.1, IC-4-0.1, and IC-8-0.1) are shown in Figure 7. Interlayered composites display a significant increase in the *R* coefficient when the frequency was increased to about 300 MHz. In turn, the *A* coefficient showed an inverse behavior to *R*, diminishing at lower frequencies, followed by slight increase (Figure 7). As observed, a higher reflection coefficient and a lower absorption coefficient was recorded as the number of layers of nonwoven 0.1 wt.% MWCNT filled carbon nanofiber mats increased. According to Equations (4)–(6), the shielding by reflection (*SE*_ref_) or reflection loss, the shielding by absorption (*SE*_abs_) or absorption loss, and the total SE (*SE*_Total_) of the composites that are mentioned above were calculated and plotted vs. frequency (Figure 8). In the 0–300 MHz frequency range, *SE*_abs_ decreases while *SE*_ref_ augments. Above 600 MHz, both *SE*_abs_ and *SE*_ref_ exhibit a constant trend as frequency increases up to 1300 MHz. All of the samples show *SE*_abs_ higher than *SE*_ref_. *SE*_abs_ represents 75, 82, and 81% of the overall shielding for the IC-1-0.1, IC-4-0.1, and IC-8-0.1 composites, respectively. Also, it is clearly observed that *SE*_abs_ increases as the number of layers increased from 1 to 4; however, in the IC-8-0.1 composite, this trend was not observed, showing almost the same absorption loss than the IC-4-0.1 composite.

Theoretical analysis of the EMI SE was carried out to evaluate the individual contributions of absorption and reflection according to [30,32,33,34,45]:

(10) $$SE_{A}=8.7\frac{t}{\delta }=8.7t\sqrt{\pi f\mu \sigma }$$

(11) $$SE_{R}=20\;log\left(\frac{\sqrt{\mu_{0}\sigma }}{4\sqrt{2\pi f\mu \varepsilon_{0}}}\right)$$

where *t* is the thickness of the sample, *f* is the frequency, *μ* is the magnetic permeability = *μ*_0_*μ*_r_ (*μ*_0_ = 4*π* × 10^−7^ H·m^−1^, *μ*_r_ = 1), *σ* is the bulk electrical conductivity in Ω^−1^·m^−1^, and *ε*_0_ = 8.854 × 10^−12^ F·m^−1^.

**Figure 9:**
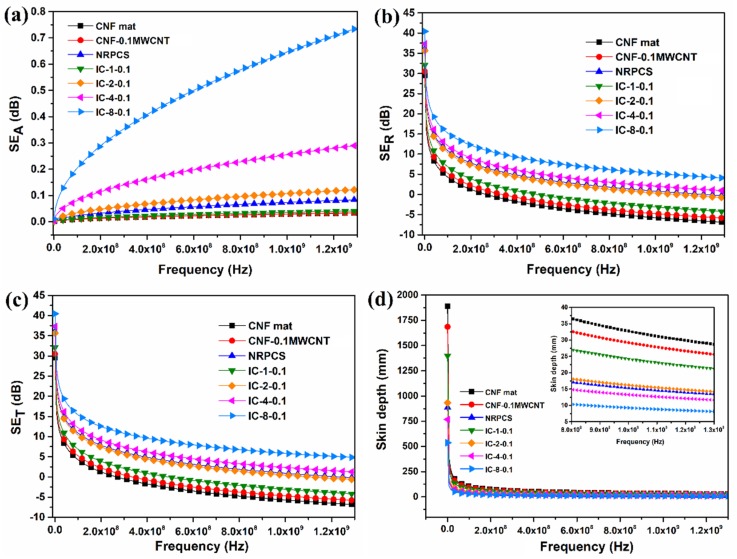
Calculated EMI SE versus frequency. (**a**) *SE*_A_; (**b**) *SE*_R_; (**c**) *SE*_T_ of CNF mat, 0.1 wt.% MWCNT filled carbon nanofiber mat, NRPCS and interlayered composites formed by different number of layers of 0.1 wt.% MWCNT filled carbon nanofiber mats; and, (**d**) Skin depth dependence with respect to frequency. The inset in (**d**) shows a zooming view of skin depth at higher frequencies (0.8–1.3 GHz).

Figure 9a–c shows the calculated EMI SE by absorption (*SE*_A_), reflection (*SE*_R_), and the total SE (*SE*_T_) of some representative samples. As observed, *SE*_A_ increases as frequency increases. In the interlayered composites, it is clearly observed that, as the number of layers (or the thickness) is increased, the SE by absorption augments (Figure 9a). This behavior is due to the larger presence of effective material, which sustains the fact that materials with higher thickness (at the scale of mm) exhibit significant absorption attenuation in EMI shielding [4,32]. On the other hand, the *SE*_R_ or reflection loss plotted in Figure 9b shows an inverse behavior to *SE*_A_, decreasing their value as the frequency increases. Interlayered composites display a higher reflection loss as the number of layers increase. Furthermore, it was found that the contribution of *SE*_R_ to the total shielding (Figure 9c) was notably higher than the contribution of *SE*_A_ in all the samples.

Besides, Figure 9a shows that, even though the absorption component increases with increasing the number of layers and the frequency, the contribution was almost negligible when compared to the reflection component (Figure 9b). Furthermore, the theoretical *SE*_T_ differed from the values that were obtained experimentally, for example, for the eight-layered system, the *SE*_T_ is calculated to be ~10 dB (Figure 9c) when it experimentally is 52 dB, as shown in Figure 6b and Table 3. These results reflect that, in this type of systems, the multiple reflection contribution seems significant. Since multiple reflections depend on the thickness relationship between skin depth and distance from reflection to interface; the skin depth of the samples was obtained from Equation (12) [4,36]:

(12) $$\delta =\frac{1}{\sqrt{\pi f\mu \sigma }}$$

As shown in Figure 9d, the skin depth of all samples diminished when the frequency was increased. In the inset, it is clearly observed that, in the materials with lower electrical conductivity and thickness, the skin depth recorded higher values; whereas, in the interlayered composites, the increase in the number of layers, the thickness, and the electrical conductivity led to notable decreases in skin depth.

## 4. Discussion

As observed, the presence of low percentages of MWCNT enhances electrical conduction along the carbon nanofiber network. Similar observations have also been reported in previous studies related to MWCNT reinforced carbon nanofibers used in hybrid or multiscale structures [15,38,46,47,48]. The infiltration process of the CNF mat by the polymer composite allowed the formation of interconnected areas and continuous paths that led to synergistic effects on the electrical properties of the interlayered composite. Given the two-dimensional (2D) structure of the nanofiber mats, there is a preferential conduction path in the in-plane direction, which does not favor the electron transport in the through-plane direction. Similar findings have been documented in previous studies of layered composites, showing decreases from one to four orders of magnitude with respect to the in-plane measurements [19,20]. The lower values that were observed in the through-plane conductivity measurements are also due to the higher number of interfaces. It is also found that the in-plane conductivity, even though increases upon the addition of layers, the effect is minimal when compared to the effect of the number of layers on the through-plane conductivity, which increases at least one order of magnitude upon the addition of more layers. 

The incorporation of MWCNT (0.05 and 0.1 wt.%) to the nonwoven CNF mats, as well as the increase in the number of layers of the interlayered composites, led to an enhancement of EMI shielding performance. Literature shows a broad number of studies that focus on the development of flexible, lightweight high-efficiency EMI shielding composites in a variety of structures (foams, solid/films or stacked/laminated materials). Table 4 summarizes some shielding effectiveness values previously reported and, for comparison, the results of the present work on similar materials are included. As a first observation, carbon-based fillers are mostly preferred to increased EMI SE performance due to their large aspect ratio and low density when compared to metal fillers. EMI SE is mostly proportional to the thickness of the samples. However, in the studies of Chang et al. [49], Raagulan et al. [43], Jou et al. [50], and Lee at al. [34], high values of EMI SE (34, 54, 60, and 133 dB, respectively) in materials, like films and foams, with relatively low thicknesses (from 0.15 to 0.6 mm) were found. In our case, the value of 52 dB obtained for the IC-8-0.1 composite is quite promising and comparable to those that were reported by these authors when considering that the thickness (0.65 mm) and amount of filler used in this interlayered composite is lower than the reported by Chang et al. [49] and Jou et al. [50].

A maximum value of 1148 dB·cm^3^·g^−1^ has been reported by Zeng et al. [41] in anisotropic porous water-borne polyurethane (WPU)-MWCNT composites assembled by the freeze-drying method. These structures exhibit low density (0.126 g·cm^−3^). On the other hand, it is observed that a value of 76.5 dB·cm^3^·g^−1^ was obtained for the interlayered composite that was developed in the current work, which surpasses the values that were obtained in various porous structures with similar thicknesses (< 1 mm), as listed in Table 4. This value demonstrates that the IC-8-0.1 composite is sufficiently a lightweight system that exhibits an adequate EMI shielding performance and it can be produced through a facile, cost-effective, and scalable method when compared to other systems.

When considering the values of absolute SE (SEE/*t*), the CNF-MWCNT mat containing 0.1 wt.% of MWCNT, developed in this work, exhibited a value of 5433.3 dB·cm^2^·g^−1^, which is higher than those of flexible graphite (606 dB·cm^2^·g^−1^) [51] and graphene foams (5000 and 1324 dB·cm^2^·g^−1^) [40,43]. In turn, the eight-layered composite showed a value of SSE/*t* slightly lower than those that were reported by Raagulan et al. [43] for MGNC-PVDF foams (1324 and 1944 dB·cm^2^·g^−1^ in the *S*-band and *X*-band region, respectively) whose thicknesses are similar to our composites.

Based on the power coefficients and the shielding mechanisms that are shown in Figure 7 and Figure 8, it can be inferred that the developed interlayered composites possess high intrinsic absorption capabilities. However, when the electromagnetic waves strike the material, the reflection phenomena take place before absorption and most of the incident wave is reflected, as previously observed by Al-Saleh et al. [31] in ABS/CNT nanocomposites. According to earlier studies, when the distance between the reflecting surfaces or interfaces (shielding thickness) is larger when compared to the skin depth, the conductive materials would attenuate internal reflections, and thus, multiple reflections could be neglected [4,32,36,39,56]. Conversely, if the shielding thickness of the materials is smaller than the skin depth, multiple reflections would be considered in EMI shielding. Therefore, based on the results that were obtained, the calculated skin depth of all the samples is larger than the shielding thickness; so, multiple reflections are not to be ignored. When the thickness of the sample was smaller than the skin depth, low SE values were observed, however, when the number of layers increased, the skin depth becomes relatively smaller, therefore, increasing the overall shielding effectiveness. These results also show that the effect of multiple reflections is significant, given the high number of interfaces and the presence of fillers.

In summary, the interlayered composites that were produced in this work represent a multifunctional system given its flexibility, lightweight, and high ability to attenuate electromagnetic waves; furthermore, when compared to other developed materials, the preparation of these composites is relatively simpler, which would facilitate their production at a large scale. 

## 5. Conclusions

Carbon hybrid nanofiber mats and nanoreinforced polypropylene composites were developed and used to fabricate interlayered composites. The developed carbon fiber mats were prepared using an industrial scalable technique, Forcespinning^®^, while the nanoreinforced polymer composite was prepared using a high shear mixer. The developed interlayered structures showed enhanced electrical conductivity and shielding of electromagnetic interference, in an eight-layer flexible composite that was composed of 18 wt.% (43 vol.%) carbon content, 12 wt.% (9 vol.%) MWCNT, and 70 wt.% (48 vol.%) of PP, the electrical conductivity was shown to be 6.1 S·cm^−1^ (0.16 Ω·cm in electrical resistivity), while the shielding effectiveness was 52 dB for a 0.65 mm thick sample. When compared to previously reported studies, the development of this interlayered system provides a highly flexible material with an ability to tailor EMI shielding needs. The developed interlayered composites possess high intrinsic absorption capabilities. However, when the electromagnetic waves strike the material, the reflection phenomena take place before absorption, most of the incident wave is reflected and the rest travels through the system. This study opens up new opportunities for the fabrication of novel lightweight materials to be used in electronics; especially in those where communication systems, such as in airplanes or satellites, could be compromised due to the overcrowding of the spectral bands, the developed material could help to prevent the degradation of system performance.

## Figures and Tables

**Table 1:** General characteristics of the developed samples.

ID Sample	CNF Mat	CNF–0.05MWCNT	CNF–0.1MWCNT	NRPCS	IC–1–0 ^2,3^	IC–1–0.05	IC–1–0.1	IC–2–0.1	IC–4–0.1	IC–8–0.1
Parameter										
Thickness (mm)	0.11	0.09	0.09	0.13	0.10	0.10	0.10	0.16	0.30	0.65
Density (g·cm^−3^)	0.33	0.30	0.28	0.99	0.79	0.79	0.78	0.73	0.70	0.68
CNF (wt.%)	100	99.9	99.9	–	12.5	11.2	10.6	13.9	16.2	17.7
CNF (vol.%)	100	99.9	99.9	–	29.8	29.5	29.6	36.4	40.7	43.3
MWCNT in CNF (wt.%)	–	0.05	0.1	15	–	6 × 10^−3^	1.1 × 10^−2^	1.4 × 10^−2^	1.6 × 10^−2^	1.8 × 10^−2^
MWCNT in CNF (vol.%)	–	0.01	0.02	7.1	–	1.4× 10^−2^	2.9 × 10^−2^	3.6 × 10^−2^	4.1 × 10^−2^	4.3 × 10^−2^
MWCNT TC ^1^ (wt.%)	–	–	–	–	13.1	13.3	13.4	13	12.6	12.4
MWCNT TC (vol.%)	–	–	–	–	10.5	10.6	10.6	9.6	8.9	8.5
PP TC (wt.%)	–	–	–	85	74.4	75.4	76	73.2	71.2	70
PP TC (vol.%)	–	–	–	92.9	60	60	60	54.1	50.4	48.2

^1^ TC—Total Content, ^2^ IC—Interlayered composite, ^3^ IC—# layers– wt.% MWCNT content in the CNF mat.

**Table 2:** Values of in-plane and through-plane electrical resistivity/conductivity of produced composites.

ID Sample	Thickness (mm)	In-Plane Resistivity, *ρ_i_* (Ω·cm)	In-Plane Conductivity, *σ_i_* (S·cm^−1^)	Through-Plane Resistivity, *ρ_t_* (Ω·cm)	Through-Plane Conductivity, *σ_t_* (S·cm^−1^)
CNF mat	0.11	0.89 ± 0.07	1.1 ± 0.1	423	2.4 × 10^−3^
CNF-0.05MWCNT	0.09	0.38 ± 0.02	2.6 ± 0.1	–	–
CNF-0.1MWCNT	0.09	0.36 ± 0.01	2.8 ± 0.1	337	3.0 × 10^−3^
NRPCS	0.13	0.67 ± 0.05	1.5 ± 0.1	93	1.1 × 10^−2^
IC-1-0	0.10	0.35 ± 0.05	2.8 ± 0.5	–	–
IC-1-0.05	0.10	0.26 ± 0.02	3.8 ± 0.2	–	–
IC-1-0.1	0.10	0.23 ± 0.02	4.5 ± 0.3	232	4.3 × 10^−3^
IC-2-0.1	0.16	0.20 ± 0.02	4.9 ± 0.4	104	9.6 × 10^−3^
IC-4-0.1	0.30	0.19 ± 0.06	5.3 ± 1.5	70	1.4 × 10^−2^
IC-8-0.1	0.65	0.16 ± 0.03	6.1 ± 1.1	34	3.0 × 10^−2^

**Table 3:** Values of average EMI SE, specific SE (SSE), and absolute shielding effectiveness (SSE/*t*) of produced composites.

ID Sample	Average EMI SE (dB)	SSE (dB·cm^3^·g^−1^)	SSE/*t* (dB·cm^2^·g^−1^)
CNF mat	11.9	36.1	3281.8
CNF-0.05MWCNT	12.2	40.7	4522.2
CNF-0.1MWCNT	13.7	48.9	5433.3
NRPCS	13.9	14.1	1084.6
IC-1-0	14.3	18.1	1810.0
IC-1-0.05	15.4	19.5	1950.0
IC-1-0.1	16.9	21.7	2170.0
IC-2-0.1	31.1	42.6	2662.5
IC-4-0.1	41.5	59.3	1976.7
IC-8-0.1	52.0	76.5	1176.9

**Table 4:** Comparison of reported values of EMI SE, SSE, and SSE/*t* for different composites and the developed materials in this work.

Material	Filler Content	*t* (mm)	EMI SE (dB)	SSE (dB·cm^3^·g^−1^)	SSE/*t* (dB·cm^2^·g^−1^)	Frequency Range (GHz)	Ref.
Flexible graphite (Grafoil)	N/A ^1^	3.1	130	118	606	1–2	[51]
Carbon-carbon matrix composite + CCF	N/A	2.4	124	N/A	N/A	0.0003−1.5	[52]
EVA-SCF ^2^ sheets	30 phr	1.8−3.5	25 34	N/A	N/A	0.1–2 8–12	[53]
LCP ^3^-CNF composites	15 wt.%	1.45	~41	N/A	N/A	0.015–1.5	[54]
LCP-CNT composites	50 wt.%	1	~60	N/A	N/A	0.3–1.8	[50]
WPU -CF-CNT film	33 wt.% 13 wt. %	0.15	34	N/A	N/A	0.05–1.5	[49]
Graphene-CNT-Fe_2_O_3_ + PEDOT:PSS ^4^ film	N/A	0.6	133	N/A	N/A	8–12	[34]
PCL ^5^-MWCNT foam	2 wt.%	20	60–80	193–258	97–129	0.04–40	[55]
MGNC ^6^ foam	PVDF ^7^	0.35 0.35	43 54	46 68	1324 1944	0–3 8.2–12.4	[43]
PDMS ^8^-graphene foam	0.8 wt.%	1	30	500	5000	8–12	[40]
PP-SSF ^9^ foam	1.1 vol.%	3.1	48	75	242	8–12.4	[42]
WPU-MWCNT foam	2.2 vol.%	4.5	52	1148	2551	8.2–12.4	[41]
PE-MWCNT BP laminates	N/A	1.5	~100	N/A	N/A	2–18	[23]
PVA-MLG ^10^ sandwich structure	60 vol.%	0.04-0.06	14	N/A	N/A	8.2–12.4	[32]
PET-CF layered composite	N/A	1.98	60	N/A	N/A	0.03–1.5	[25]
CNF-MWCNT mat IC-8-0.1 interlayered composite	0.1 wt.% 12 wt.%	0.09 0.65	13.7 52	48.9 76.5	5433.3 1176.9	0.0003–1.3	This work

^1^ N/A—value not reported or not available enough data to calculate it, ^2^ EVA-SCF—Ethylene-vinyl acetate co-polymer-short carbon fiber, ^3^ LCP—liquid crystal polymer, ^4^ PEDOT:PSS—poly(3,4-ethylenedioxythiophene) poly(4-styrenesulfonate), ^5^ PCL—polycaprolactone, ^6^ MGNC—Mxene-graphene composite, ^7^ PVDF—polyvinylidene fluoride, ^8^ PDMS—poly(dimethyl siloxane), ^9^ SSF—stainless steel fibers, ^10^ MLG—multilayer graphene.
